# Movements of marine and estuarine turtles during Hurricane Michael

**DOI:** 10.1038/s41598-021-81234-3

**Published:** 2021-01-15

**Authors:** Margaret M. Lamont, Darren Johnson, Daniel J. Catizone

**Affiliations:** 1 U.S. Geological Survey, Wetland and Aquatic Research Center, Gainesville, FL 32653 USA; 2 Cherokee Nations Systems Solutions, Contracted to U.S. Geological Survey, Wetland and Aquatic Research Center, Lafayette, LA 70506 USA

**Keywords:** Animal behaviour, Herpetology, Animal migration, Ecology, Zoology

## Abstract

Natural disturbances are an important driver of population dynamics. Because it is difficult to observe wildlife during these events, our understanding of the strategies that species use to survive these disturbances is limited. On October 10, 2018, Hurricane Michael made landfall on Florida’s northwest coast. Using satellite and acoustic telemetry, we documented movements of 6 individual turtles: one loggerhead sea turtle, one Kemp’s ridley sea turtle, three green sea turtles and one diamondback terrapin, in a coastal bay located less than 30 km from hurricane landfall. Post-storm survival was confirmed for all but the Kemp’s ridley; the final condition of that individual remains unknown. No obvious movements were observed for the remaining turtles however the loggerhead used a larger home range in the week after the storm. This study highlights the resiliency of turtles in response to extreme weather conditions. However, long-term impacts to these species from habitat changes post-hurricane are unknown.

## Introduction

Natural disturbances can impact animal populations in many ways including altering community structure^1^, displacing individuals^2^, reducing reproductive output^3^, lowering individual fitness^4^, and changing movement patterns^5^. In addition, these events can alter habitat and environmental conditions, which can create suboptimal conditions for organisms. Because it is difficult to observe wildlife during these events, our understanding of the strategies that species use to survive or mitigate these disturbances is limited^6–10^. This knowledge gap hampers our ability to identify optimal conditions required by imperiled species for survival during extreme events.

On October 10, 2018, Hurricane Michael made landfall near Tyndall Air Force Base on Florida’s northwest coast. As a Category 5 storm with maximum winds greater than 140 kts, Hurricane Michael was the third most intense storm to make landfall in the contiguous United States^11^. Storm surge was estimated at 9–14 ft in Bay and Gulf Counties, Florida^11^ and the combination of water and wind resulted in the destruction of man-made structures and natural habitats. An estimated 39% of longleaf pine habitat in Florida showed immediate impacts from the storm^12^ and salinity of wetlands in the region was altered^13^. Marine habitats may also have been impacted, which could have negative consequences for the species that rely on them for survival^14,15^.

Northwest Florida’s estuarine and marine systems are rich in natural resources. The dominant feature of the region is the Apalachicola River, which has the greatest volume of all Florida’s rivers^16^. The nutrients deposited by the river sustain a high biological diversity throughout the region including the highest species density of amphibians and reptiles on the continent north of Mexico^17^. These reptiles include a variety of turtle species that inhabit estuarine and marine habitats such as diamondback terrapins (*Malaclemys terrapin*) and loggerhead (*Caretta caretta*), Kemp’s ridley (*Lepidochelys kempii*) and green sea turtles (*Chelonia mydas*).

**Figure 1:**
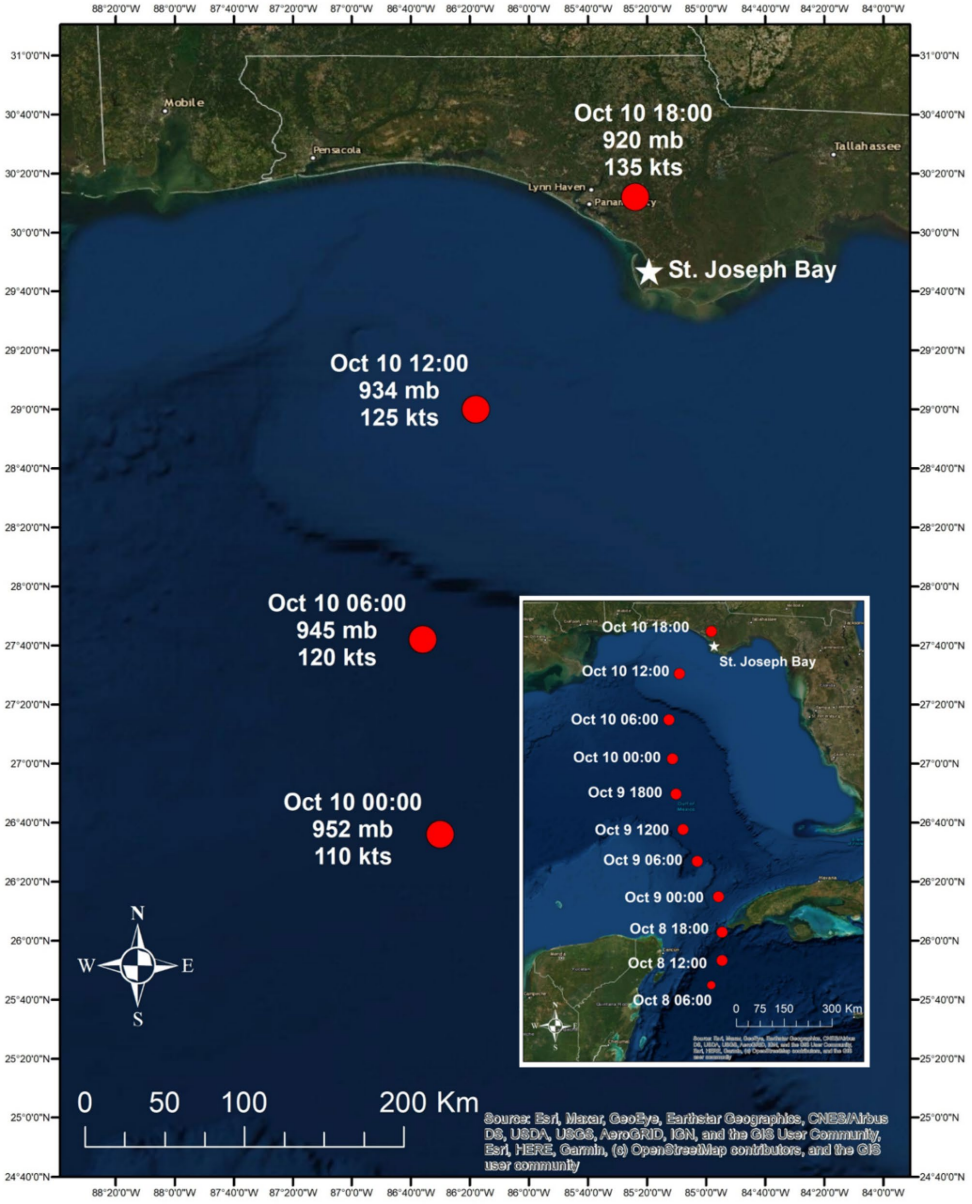
Hurricane Michael made landfall on October 10, 2018 approximately 15 km west of St. Joseph Bay, Florida where marine and estuarine turtles were being tracked using satellite and acoustic telemetry (hurricane data from Bevan et al. 2019). This map (with self-created symbols/shapes/text) was created using ESRI (Environmental Systems Resource Institute; http://www.esri.com/software/arcgis) ArcMap software, version 10.7.1. Basemap sources include: ESRI, Maxar, GeoEye, Earthstar Geographics, CNES/Airbus DS, USDA, USGS, AeroGRID, IGN, and the GIS User Community. Hurricane data contributed by Beven et al. (2019).

This area is also susceptible to hurricane impacts. Since hurricane records have been kept, the coasts of Mississippi, Alabama and Northwest Florida have had direct hits by more than one-third of the 273 storms that have affected the US mainland^18^. Hurricanes can displace freshwater and terrestrial reptiles^19,20^ and cause direct mortality of many species^20–22^. Understanding the conditions needed for species survival during hurricanes in this region is critical to population recovery. Using satellite and acoustic telemetry, we documented movements of four turtle species as Hurricane Michael moved through Northwest Florida (Fig. 1).

## Results

Hurricane Michael made landfall approximately 30 km west of SJB on October 10, 2018 at approximately 13:30 local time [Eastern Daylight Time (ET); 17:30 UTC; Fig. 1]. Barometric pressure at landfall was estimated at 919 mb and maximum sustained winds were from the southwest (from approximately 210°) at 140 kts^11^. The National Weather Service suggested the storm was still intensifying as it made landfall^11^. Barometric pressure dropped from 1133 mb on October 1 to 1020 mb on October 2 and then again to 1014 mb on October 8 as the storm entered the southern GoM^11^ (see Fig. 1). One loggerhead (July 27, 2018), one Kemp’s ridley (September 26, 2018) and one diamondback terrapin (July 10, 2018) were captured and satellite tagged in SJB prior to the hurricane (Table 1). All three individuals were carrying active satellite tags during the storm. In addition, three green turtles were captured and tagged on October 4, 2018 and were carrying active acoustic tags during the storm (Table 1). Using size as an estimator of life-stage^23–25^, the loggerhead was a large juvenile, the Kemp’s ridley was either a large juvenile or an adult female, and the green turtles were juveniles. The terrapin was an adult female. Because terrapins are highly exploited in the commercial pet trade^26,27^, and providing location data through publication of research results can direct poachers to capture sites^28^, we chose not to present specific location data for the terrapin tracked during this study. We believe that information is not necessary to understand the movement patterns of this individual during the storm.

**Table 1:** Capture, tag transmission and size information for turtles tracked in Northwest Florida during Hurricane Michael in October 2018.

Capture	First loc/detect	Last loc/detect	Species	Size (cm)	Tag type
7/10/2018	7/10/2018	12/3/2018	Terrapin	18.9	Satellite
7/27/2018	7/27/2018	10/8/2019	Loggerhead	76.5	Satellite
9/26/2018	9/28/2018	10/10/2018	Kemp's ridley	54.6	Satellite
10/4/2018	10/11/2018	6/22/2019	Green	40.3	Acoustic
10/4/2018	10/4/2018	5/17/2019	Green	39.0	Acoustic
10/4/2018	10/5/2018	5/14/2019	Green	34.0	Acoustic

*Capture* date of capture and tagging, *first loc/detect* date the first location or detection from the tag was documented, *last loc/detect* date the last location or detection from the tag was documented, *species* turtle species, *size* straight carapace length in cm, *tag type* whether the individual was tracked via satellite or acoustic tag.

The last location received for the Kemp’s ridley was on October 10, 2018 at 22:26 ET which is approximately 9.0 h after hurricane landfall (Table 1). The last location received from the terrapin was on December 3, 2018 which is 56 days after landfall. The loggerhead’s tag continued transmissions until July 2019 and the green turtles were still being detected > 6 months after hurricane landfall.

### Locations during landfall

**Figure 2:**
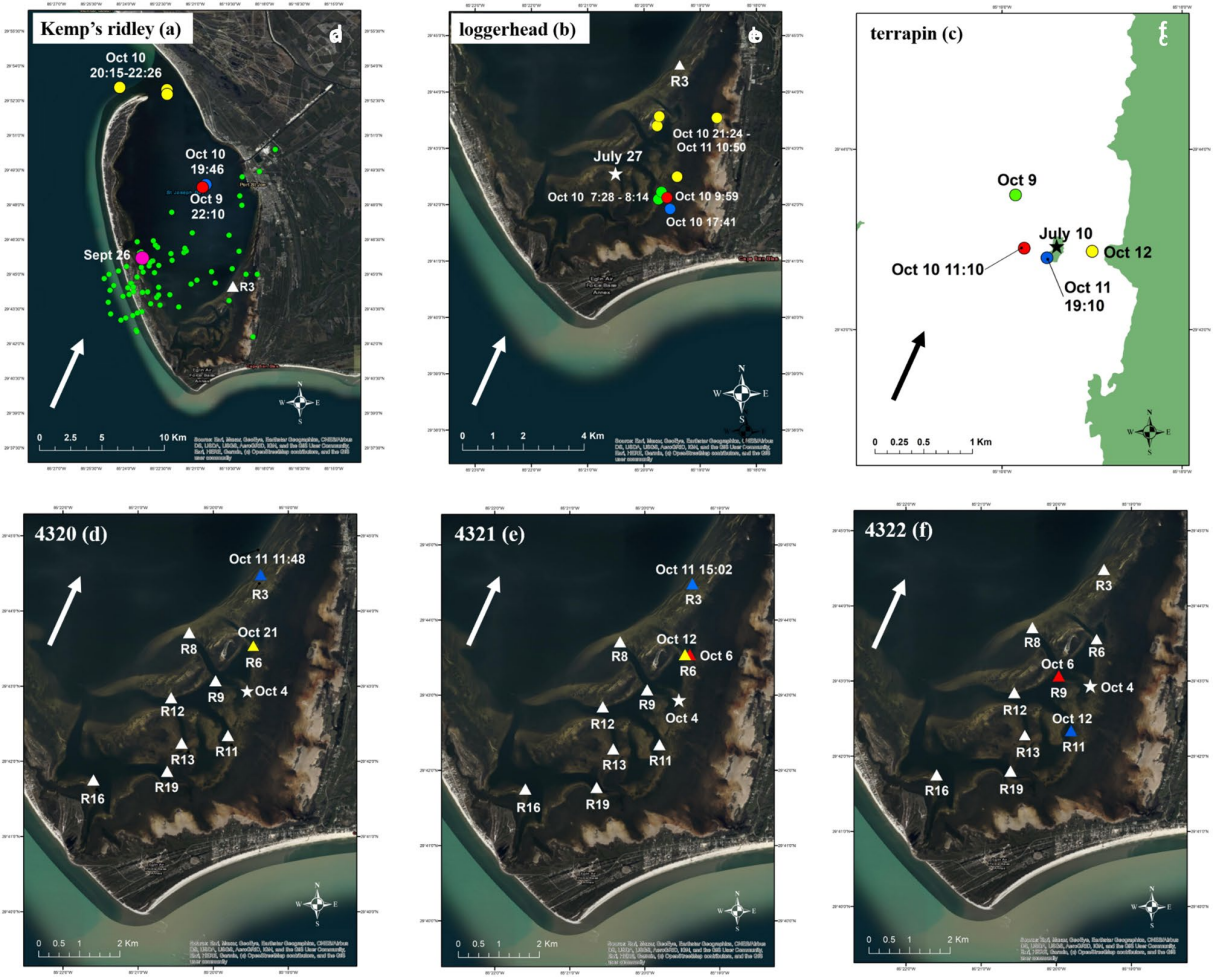
Raw locations/detections for a Kemp’s ridley (**a**), loggerhead (**b**) and diamondback terrapin (**c**) that were carrying satellite tags and three green turtles (**d**–**f**) that were carrying acoustic tags in St. Joseph Bay, Florida when Hurricane Michael made landfall at approximately 14:00 h (EDT) on October 10, 2018 in Northwest Florida. The star represents the capture location (with capture date) for each turtle (except for the Kemp’s ridley which is marked by a pink circle). Triangles (regardless of color) show locations of acoustic receivers (R# is the receiver identifier). The location of receiver 3 (R3) is included in the Kemp’s ridley and loggerhead map for reference. Green circles represent locations/detections in the days prior to the storm; all previous locations (except filtered locations; see Methods) for the Kemp’s ridley are included. Red icons represent the last available location/detection for each turtle immediately prior to hurricane landfall. Blue icons represent the first available location/detection available for each turtle immediately after hurricane landfall. Yellow icons represent locations/detections in the day(s) after landfall occurred. Dates and times (in ET) for these locations/detections are also included. The arrow indicates wind direction during hurricane landfall. Because terrapins are an exploited species, and specific location data would reveal capture locations, we chose not to display these details on a satellite image. These maps (with self-created symbols/shapes/text) were created using ESRI (Environmental Systems Resource Institute; http://www.esri.com/software/arcgis) ArcMap software, version 10.7.1. Basemap sources for all Figures, except c (terrapin), include: Esri, Maxar, GeoEye, Earthstar Geographics, CNES/Airbus DS, USDA, USGS, AeroGRID, IGN, and the GIS User Community. The basemap source for Figure c is the Florida Fish and Wildlife Research Institute (https://myfwc.com/research/gis/). Hurricane data contributed by Beven et al. (2019).

Locations were received from the loggerhead’s tag approximately 5 h before, 2 h before and 6 h after landfall (Fig. 2). The Kemp’s ridley tag transmitted approximately 21 h before and 6 h after landfall. Three additional locations were then received 7–9 h after landfall; those three locations represent the final transmissions from the tag. The terrapin tag transmitted 4 h before and 27 h after landfall.

Although tagged on October 4, the first detection of green turtle 4320’s tag was approximately 19 h after hurricane landfall (Fig. 2). Green turtle 4321’s tag was detected before hurricane landfall on October 6 and then approximately 23 h after landfall. Green turtle 4322’s tag was also detected before landfall on October 6 but was not detected again until 47 h (October 12) after landfall.

### Home ranges

**Figure 3:**
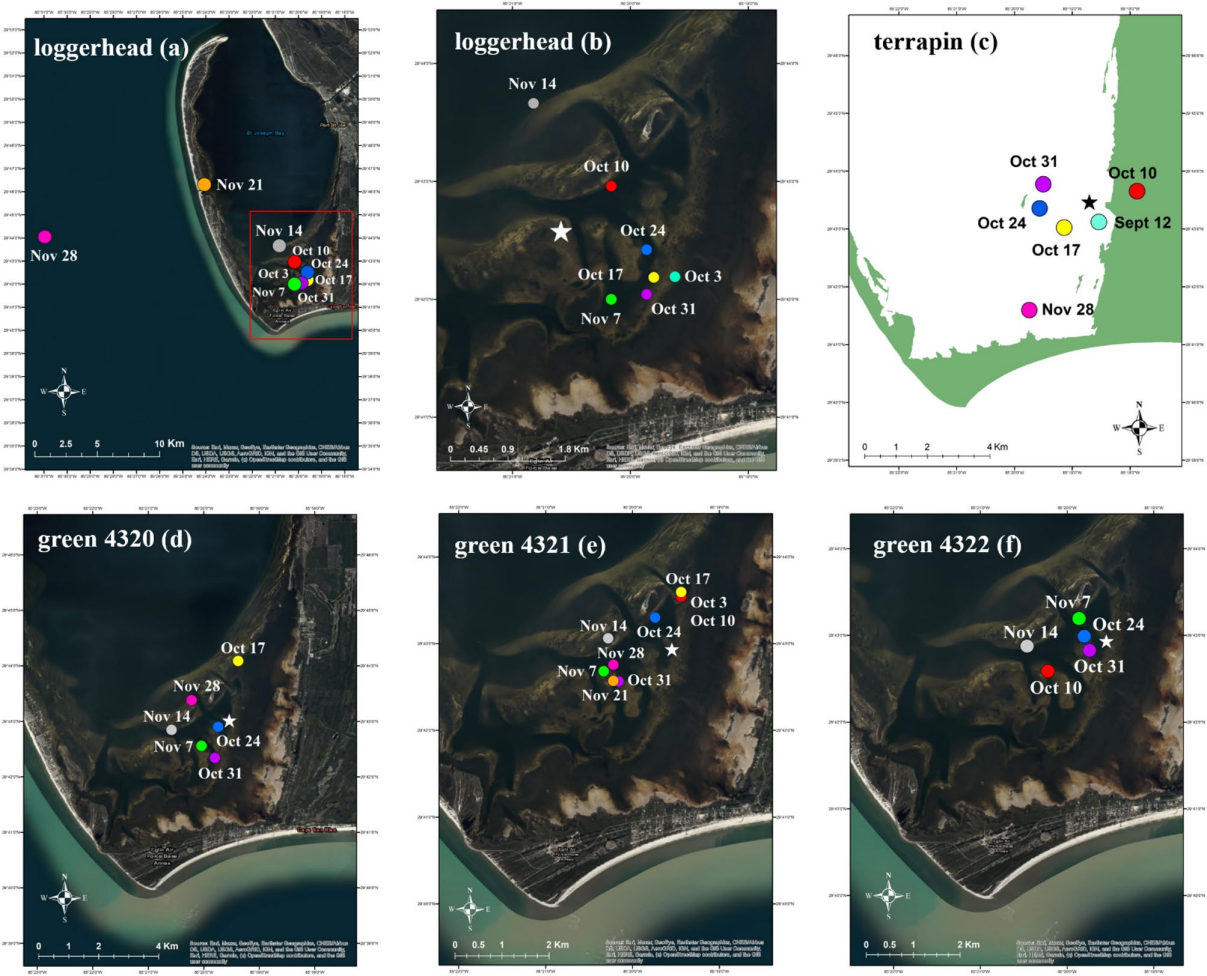
Weekly home range centroids for turtles carrying a satellite tag [loggerhead (**a**, **b**), terrapin (**c**); Minimum Convex Polygon] or an acoustic tag [green turtles (**d**–**f**); Kernel Density Estimation] during Hurricane Michael in St. Joseph Bay, Florida. Due to a lack of data, home range maps are not provided for the Kemp’s ridley. The storm made landfall on October 10, 2018; the centroid for the week of October 10 (October 10–16) is shown by a red dot. All weekly centroids for the loggerhead are shown on the left (**a**) and a close-up of loggerhead centroids on the right (red square; shown in **b**) immediately before, during and after the storm. Capture locations for each individual are denoted with a star. If weekly centroids are not included on the map, it is due to gaps in data. Because terrapins are an exploited species and providing exact locations for individuals may contribute to poaching, we chose not to display locations on satellite imagery. These maps (with self-created symbols/shapes/text) were created using ESRI (Environmental Systems Resource Institute; http://www.esri.com/software/arcgis) ArcMap software, version 10.7.1. Basemap sources for all Figures, except c (terrapin), include: Esri, Maxar, GeoEye, Earthstar Geographics, CNES/Airbus DS, USDA, USGS, AeroGRID, IGN, and the GIS User Community. The basemap source for Figure c is Florida Fish and Wildlife Conservation Commission-Fish and Wildlife Research Institute (https://myfwc.com/research/gis/).

Home range centroids for the loggerhead, terrapin and green turtle 4321 for the week immediately after hurricane landfall (Oct 10) were located north or northeast of the turtle’s original capture location (Fig. 3). The centroid for green turtle 4322 was located southwest of the turtle’s capture location and green turtle 4320 had no data available for that week.

Home range sizes varied among individuals (Table 2) with the largest home range exhibited by green turtle 4320 (mean 4.45; SD 4.330; range 0.004–11.180) and the smallest by the terrapin (mean 0.525; SD 0.867; range 0.003–3.191). Home range sizes also varied among weeks for each individual (Supplemental Figure 1). The loggerhead used a larger home range the week after the storm than in all of the previous weeks (but not subsequent weeks; see Discussion). For the remaining turtles, there were no obvious changes in home range sizes in the weeks immediately before or after Hurricane Michael (Table 2).

**Table 2:** Weekly home range sizes (km^2^) for a satellite-tagged loggerhead, Kemp’s ridley and diamondback terrapin (MCPs), and three acoustic-tagged green turtles (KUDs) in St. Joseph Bay, Florida in 2018.

	Loggerhead	Kemp's ridley	Terrapin	Green 4320	Green 4321	Green 4322
7/18	–	–	0.105	–	–	–
7/25	–	–	0.281	–	–	–
8/1	–	–	–	–	–	–
8/8	–	–	0.346	–	–	–
8/15	0.155	–	0.733	–	–	–
8/22	0.180	–	0.062	–	–	–
8/29	0.022	–	–	–	–	–
9/5	0.175	–	–	–	–	–
9/12	0.096	–	0.110	–	–	–
9/19	0.057	–	–	–	–	–
9/26	0.179	2.963	–	–	–	–
10/3	0.093	0.983	–	–	1.578	–
*10/10*	*0.548*	*–*	*0.236*		*0.010*	*1.578*
10/17	0.042	–	0.571	4.606	0.876	0.010
10/24	0.190	–	0.231	7.387	11.989	0.876
10/31	0.033	–	0.427	0.066	1.213	11.989
11/7	0.065	–	–	3.432	0.777	1.213
11/14	1.215	–	–	0.004	1.339	0.777
11/21	9.483	–	–	11.177	4.464	–
11/28	7.453	–	0.003	–	2.116	–
Mean	1.160	1.973	0.302	4.445	2.707	2.741
SD	2.700	1.400	0.220	4.334	3.697	4.561

The date in the first column represents the start of the week (e.g. 7/18 = July 18—July 24). Category 5 Hurricane Michael made landfall in Northwest Florida on October 10, 2018 and the week after storm landfall (October 10 – October 16) is highlighted in italics.

## Discussion

The long-term effects of Hurricane Michael on Northwest Florida habitats are still unknown. Despite the record-breaking winds and extreme storm surge, tags on five of the six turtles tracked during landfall continued transmissions post-storm thereby confirming turtle survival. There are multiple strategies that turtles utilize to survive during and after extreme weather events including altering dive behavior, increasing home range size and shifting habitat use^29–31^. These studies have typically involved larger samples sizes^30^ or have documented long-distance movements^31^. The turtles tracked in SJB during Hurricane Michael showed no large-scale movements or changes in behavior in response to the storm. Individual variation in hurricane response for hawksbill sea turtles^30^, and other reptiles such as alligators^32^ has been documented. This includes some individuals that show no obvious response^32^. Our results suggest turtles in SJB responded little, at least with obvious and/or large-scale movements, to the storm both before and after landfall. Our small sample sizes, paired with limitations of non-GPS capable satellite tags in identifying fine-scale movements^33^ and the potential impacts of shallow and rough water on acoustic receiver detections^34^, make it difficult for us to confirm fine-scale changes in behavior. In addition, it is difficult to say if behaviors observed in this study (e.g. larger home range used by the loggerhead after the storm) were in response to the storm or some other factor. We recognize these limitations however, we agree with Sergio et al.^35^ that “it is ever more urgent and fundamental to increase our knowledge of species responses to disturbance, especially to predict potential future responses by endangered organisms”.

### Kemp’s ridley

Because the satellite tag on the Kemp’s ridley stopped transmitting approximately 5 h after hurricane landfall, the fate of this individual is unknown. There are many reasons why satellite tags stop transmitting including tag malfunction (e.g. salt-water switch), battery life, antenna damage or animal mortality^36^. The satellite tag was deployed on the Kemp’s ridley in SJB only two weeks before the hurricane and outputs from the tag showed strong battery, no signs of biofouling, and no salt-water switch failures. When sea turtles die, they quickly sink^37^. The abrupt cessation of tag transmissions soon after landfall could indicate mortality or tag damage. Unfortunately, it is difficult to discriminate between those two possible events^36^.

Typically, juvenile Kemp’s ridleys inhabit nearshore, shallow waters^38–40^. From 2013 to 2017, seven juvenile Kemp’s ridleys were tracked in SJB; those individuals established home ranges that were within 1.5 km of shore and in relatively shallow water (mean 3.6 m). In the weeks prior to hurricane landfall, a Harmful Algal Bloom (HAB) of the toxic dinoflagellate *Karenia brevis* impacted the northwest Florida coast (https://oceanservice.noaa.gov/hazards/hab/florida-2018.html). Termed “red tide” events, these HAB’s cause disproportionate mortality to Kemp’s ridleys as compared to green turtles and loggerheads and exposure to brevetoxins can result in neurological impairment in sea turtles^37,41^. This HAB may have impacted the Kemp’s ridley’s behavior during our tracking period.

### Loggerhead

The loggerhead we tracked during Hurricane Michael appeared to remain in relatively shallow water throughout the storm and was near the water’s surface within two hours of hurricane landfall. Loggerheads are strong swimmers and can maintain their position despite high winds and storm currents^42–45^ however our results suggest that even in Category 5 winds, loggerheads are able to not only survive but to remain in place in relatively shallow water.

In the week after the storm, the loggerhead used a larger home range that included deep water north of her capture location (see Supplemental Figure S1). Short-term (i.e. weekly) expansions in space-use following environmental perturbations have been documented for many species^35^ including hawksbill sea turtles^30^ and may reflect increased foraging effort required after disturbances or use of new resting locations^30^. The expanded area used the week after the storm was also used by the loggerhead during the weeks of November 14 and 21. At this time, the loggerhead began moving north and by the end of November, the turtle was out of SJB. These November movements were in response to falling temperatures, which have been previously documented for loggerheads in SJB^46^. Mean air temperatures [recorded at the National Oceanographic and Atmospheric Administration’s weather buoy (APCF1) located approximately 30 km east of SJB], dropped from 21.5 °C (Nov 8–14), to 17.6 °C (Nov 15–21) and then even further to 13.8 °C (Nov 22–28) during this time period. It is well reported that loggerheads use deep water as a thermal refuge^46–48^ and this expanded area may have also served as a post-storm refuge for the loggerhead immediately after Hurricane Michael.

### Terrapin

The diamondback terrapin is the smallest of all species tracked during the hurricane however this individual did not appear to be impacted during the storm’s passage. While displacement of aquatic turtles, such as wood turtles (*Glyptemys insculpta*) and Johnstone River snapping turtles (*Elseya irwini*)*,* has been documented during extreme flooding, the majority of individuals in these studies were not impacted by the floods^20,49,50^. It has been suggested that individuals bury themselves or remain on the bottom in deep water to avoid extreme surface currents^20^ and this may have been the strategy utilized by the terrapin in SJB. We received a high-quality location (LC 3) approximately 2.5 h before and then again 18 h after hurricane landfall, which suggests the terrapin was at the water’s surface or on land at those times. Many turtles, including terrapins, are able to remain submerged for long periods of time through aquatic respiration or regulation of metabolism^51,52^. It is likely the terrapin in SJB remained submerged or buried through hurricane landfall to avoid displacement from winds and currents.

The week after the storm however the terrapin’s home range centroid was approximately 1.5 km east (i.e. landward) of any other weekly centroid we calculated, but this did not correspond to an increase in home range size. Although reptiles are occasionally displaced by storms those dislocations are typically farther (i.e. tens of kilometers) than the small distance (~ 1.5 km) observed here^2,19,49^. Flooding from SJB eastward was severe during and immediately after Hurricane Michael^11^ and this may have pushed the terrapin landward. As flood waters receded, she could have returned to her usual location near her capture site where she was documented the following week (October 17). However, this small shift in centroid location may also simply reflect variability in Argos locations. Regardless of whether the terrapin moved after landfall, terrapins in SJB did not appear to suffer significant consequences from the storm. After several hurricanes impacted the Florida Keys, few mangrove terrapins (*Malaclemys terrapin rhizophorarium*) were captured and it took several years for terrapins to return^53^; whereas, 6 days after Hurricane Michael, we captured two adult male terrapins in SJB, and within three weeks we captured an additional 17 individuals, including 6 females.

### Green turtles

None of the tagged green turtles were detected on any receivers for several days immediately before and during the storm. We had acoustic receivers placed throughout most of the southern end of the bay, although receivers were spaced far enough apart (ex: 1200 m in some cases) where gaps were available. Therefore, the tagged green turtles could have either remained in the southern end of the bay but in undetectable areas or moved out of the array during this time period. Unfortunately, we do not have the data to answer this question.

Both of the green turtles that were detected the day after the storm (October 11; 4320 and 4321) were located at the most northerly receiver (R3). Perhaps the green turtles moved north into deeper waters where they were undetected. Similar to loggerheads, green turtles in SJB use deep water as a thermal refuge during cold temperatures^46,54^ and these same areas may have also been used as a refuge during Hurricane Michael^30^. Again, without acoustic detections during the storm, it is impossible to know for sure how these individuals responded. After the storm passed, the turtles traveled back to their original capture location, with turtles 4321 and 4322 arriving by October 12 and turtle 4320 arriving by October 21.

Interestingly, Matley et al.^30^ reported that larger hawksbills did not shift habitat use after Hurricanes Irma and Maria. In our study, the green turtles (range 34.0–40.3 cm SCL) were much smaller than the loggerheads (76.5 cm SCL) however none of these individuals displayed obvious shifts in habitat use. Larger individuals may be more resilient to environmental perturbations^30,55^ due to increased energy reserves, competition or simple physical strength. For example, a 150 kg loggerhead may be less susceptible to dislocation from storm surge than a 6 kg green turtle. In SJB, not even the terrapin, which was the smallest turtle we tracked during the storm (0.98 kg), was clearly impacted by the storm. However, terrapins, unlike sea turtles, are able to bury themselves completely in response to environmental perturbations. It has been suggested that sea turtles bury themselves in sediment during periods of extreme cold^56^ but it is more likely that turtles simply rest on the seafloor for extended periods rather than actually bury themselves^47,57^. The ability of terrapins to utilize hibernacula as a refuge during extreme weather conditions most likely explains the lack of obvious impacts we observed for the small terrapin during Hurricane Michael.

Although we have confirmation that most of the turtles we tracked during the storm survived, long-term impacts from the hurricane may still affect these individuals^20^. Population declines in various species, including aquatic turtles, have been linked to habitat alterations caused by hurricanes^58,59^. Seagrass and salt marsh habitat used by loggerheads, Kemp’s ridleys, green turtles and terrapins may be altered by storms, although these impacts appear to be site- or storm-specific^60,61^. Seagrass habitat in Alabama was unaffected by Hurricane Katrina whereas habitat in the Florida Keys experienced significant damage from Hurricane Georges^60^. Challener et al.^61^ found no post-storm impacts to seagrass-associated, sea urchin populations in SJB immediately after Hurricane Michael and reported no evidence that the seagrass was impacted. The results of our opportunistic study highlight the resiliency of marine and estuarine turtles^62,63^. More research on hurricane response by marine and estuarine turtles is needed, particularly for Kemp’s ridleys as the individual in our study was the only turtle that may not have survived.

## Methods

### Turtle tagging

St. Joseph Bay (SJB), located in Northwest Florida in the northern Gulf of Mexico (GoM; Fig. 1), covers approximately 26,000 ha and is surrounded by mostly undeveloped land and the small town of Port St. Joe (population 3500^64^). It has a mean depth of 7 m^64^. Seagrass beds cover approximately one-sixth of the bay (4000 ha) and are most abundant in the southern end^64^. Barometric pressure, wind speed and direction data were downloaded from NOAA C-MAN station (APCF1; https://www.ndbc.noaa.gov/station_page.php?station=APCF1) located approximately 32 km east of St. Joseph Bay.

Turtles were captured in SJB from July through October 2018 (Table 1) using a set net (green turtle) or by hand (terrapin, loggerhead, Kemp’s ridley). All sea turtles were individually marked with a metal Inconel tag placed along the trailing edge of each front flipper and a Passive Integrated Transponder (PIT) tag placed subcutaneously in the left shoulder. The terrapin received a PIT tag inserted behind the left bridge, and its carapace was notched. All turtles were measured using two methodologies: (1) straight carapace length (SCL) and width (SCW) using calipers and (2) curved carapace length (CCL; measured from the nuchal notch to the tip of the left pygial scute) and width (CCW) using a cloth tape measure. Turtles were also weighed using a hand-held Pesola (Kapuskasing, Ontario, Canada) spring scale.

All turtle capture, handling and sampling methods were carried out in accordance with relevant guidelines and regulations described by the National Marine Fisheries Service (permit #17183) and Florida Fish and Wildlife Conservation Commission (permits #094, #33447). All experimental protocols were approved by USGS Institutional Animal Care protocols (Protocol USGS/WARC/GNV 2019-15; USGS/WARC/GNV 2018-04).

Satellite tags were fitted to the anterior portion of the carapace using a cool-setting epoxy (Superbond; FCGE Fiberglass Coatings, Inc, St. Petersburg, Florida) and following established protocols^65^. The terrapin received a SPOT-387 tag, the loggerhead received SPOT-287 tag, and the Kemp’s ridley was carrying a SPLASH10 tag (Wildlife Computers, Redmond, WA). We ensured that the total weight of the satellite tag and epoxy on all turtles did not exceed 5% of the individual’s body weight. Prior to satellite tag attachment, we removed epibionts, sanded the attachment site and cleaned it with isopropyl alcohol. We streamlined the attachment materials to reduce drag on the turtle^66^. All tagged turtles were released at or near their capture location. Each tag was set to be active for 24 h d^−1^.

In July 2018, we placed nine VR2W acoustic receivers (Innovasea; Bedford, Nova Scotia, Canada) throughout the southern portion of SJB (see Fig. 2). Receivers were attached to permanent channel markers using plastic electrician zip-ties. Green turtles were captured on October 4, 2018 and fitted with V13 coded acoustic transmitters (Innovasea; 6 g in water; 13 mm diameter × 36 mm length) on the right rear marginal scute of the carapace. Acoustic transmitters were attached with stainless steel wire fitted through two 3-mm diameter holes drilled in the marginal scutes. Location of transmitters did not interfere with flipper movements. A small amount of epoxy was placed under and then over the transmitter for additional adhesion. Receivers were downloaded after the storm on December 5, 2018.

### Data analysis

We calculated home ranges, along with home range centroids, for each turtle on a weekly basis. Weekly analyses were chosen based on data availability and following Matley et al.^30^. Satellite data were filtered by location class prior to analyses. We included location classes (LC) 3, 2, 1, 0, A, and B and rejected LC Z for which no error estimation was available. Argos assigns accuracy estimates of 250 m for LC 3, 250 to 500 m for LC 2, 500 to 1500 m for LC 1, and 1500 m for LC 0^67^. The estimated accuracy is unknown for LCs A and B (but these LCs can be useful; see^68,69^), and locations failing the Argos plausibility tests are tagged as class LC Z. Kalman-filtering of location data was performed by Argos. Kalman-filtering algorithm provides more estimated positions and significantly improves position accuracy, most significantly for locations obtained in LCs A and B^70^.

We used State Space Modeling (SSM) approach to calculate home ranges (but not estimate behavioral state), following Hart et al.^33^. We applied a model used by Breed et al. ^71^, which is a modified version of a model described by Jonsen et al.^72^ that estimates model parameters by Markov Chain Monte Carlo (MCMC) using JAGS via the software program R. We fit the model to data for each individual turtle to estimate location every day from five independent and parallel chains of MCMC samples. Our samples from the posterior distribution were based on 3,000 samples after a burn-in of 7000 samples and thinning the remaining samples by twenty. If convergence wasn’t reached, we increased the sample size to 6,000. We used the psrf < 1.1 as a measure of convergence.

Home ranges were calculated for all individuals using the SSM location data during each week using Multiple Convex Polygon (MCP)^70,73^ or Kernel Density Estimation (KDE)^72,74^ in R (package adehabitatHR^75^) depending on data availability. For individuals that had between 5 and 19 locations MCP was used whereas KDE was used for individuals with ≥ 20 locations. Weeks were defined based on hurricane landfall (October 10, 2018 at approximately 13:30 EDT). Hence the first week before the hurricane ranged from October 3 to October 9 and all preceding and subsequent weeks followed (e.g. week after the hurricane October 10–October 16).

In ArcMap (v10.7.1) we plotted turtle locations to document movements in the days surrounding hurricane landfall. We plotted the following locations, depending on data availability: (1) the day(s) prior to landfall, (2) the most recent location immediately before landfall, (3) the most recent location immediately after landfall and (4) the day(s) after landfall. For the Kemp’s ridley, we plotted all available locations after filtering out obvious erroneous locations. Erroneous locations were defined as those that were > 2 km into the GoM or on land^76^.

We calculated weekly home range size (km^2^) and home range centroids for the temporal extent possible for each individual. We mapped centroid locations for the week immediately prior to landfall (October 3) through November 28. If data were not available for a turtle for that entire time period, we used the temporal extent of available data. We chose November 28 as the cut off for mapping centroid locations because sea turtles in SJB typically move out of the bay and into deeper waters in the GoM in mid to late November^46^. Terrapins also become less active and presumably enter hibernacula when temperatures drop^77^ although data on over-wintering behavior by terrapins in Northwest Florida are lacking.

## Supplementary Information

Supplementary Figure.
