# Supplementary material for: Movements of marine and estuarine turtles during Hurricane Michael
Source: Sci Rep. 2021 Jan 15;11:1577. doi: 10.1038/s41598-021-81234-3 (PMC7810867; doi:10.1038/s41598-021-81234-3)
Supplement: Supplementary file 1 — Supplementary Figure. [file 41598_2021_81234_MOESM1_ESM.docx]

Movements of marine and estuarine turtles during Hurricane Michael

Margaret Lamont^1*^

Darren Johnson^2^

Daniel Catizone^1^

**Author affiliations**:

^1^ U.S. Geological Survey, Wetland and Aquatic Research Center, Gainesville, FL 32653, USA

^2^ CNT, contracted to U.S. Geological Survey, Wetland and Aquatic Research Center, Lafayette, LA, 70506, USA

^*^correspondence to: mlamont@usgs.gov

Supplemental Figure S1. Weekly home ranges for a loggerhead (a shows all home ranges while b is close-up of the area shown in the checked box) and terrapin (c) that were carrying a satellite tag, and (d-f) for green turtles that were carrying acoustic tags in St. Joseph Bay, Florida during Hurricane Michael in October 2018. These maps (with self-created symbols/shapes/text) were created using ESRI (Environmental Systems Resource Institute; http://www.esri.com/software/arcgis) ArcMap software, version 10.7.1. Basemap sources for all Figures, except c (terrapin), include: Esri, Maxar, GeoEye, Earthstar Geographics, CNES/Airbus DS, USDA, USGS, AeroGRID, IGN, and the GIS User Community. The basemap source for Figure c is the Florida Fish and Wildlife Conservation Commission-Fish and Wildlife Research Institute (https://myfwc.com/research/gis/).


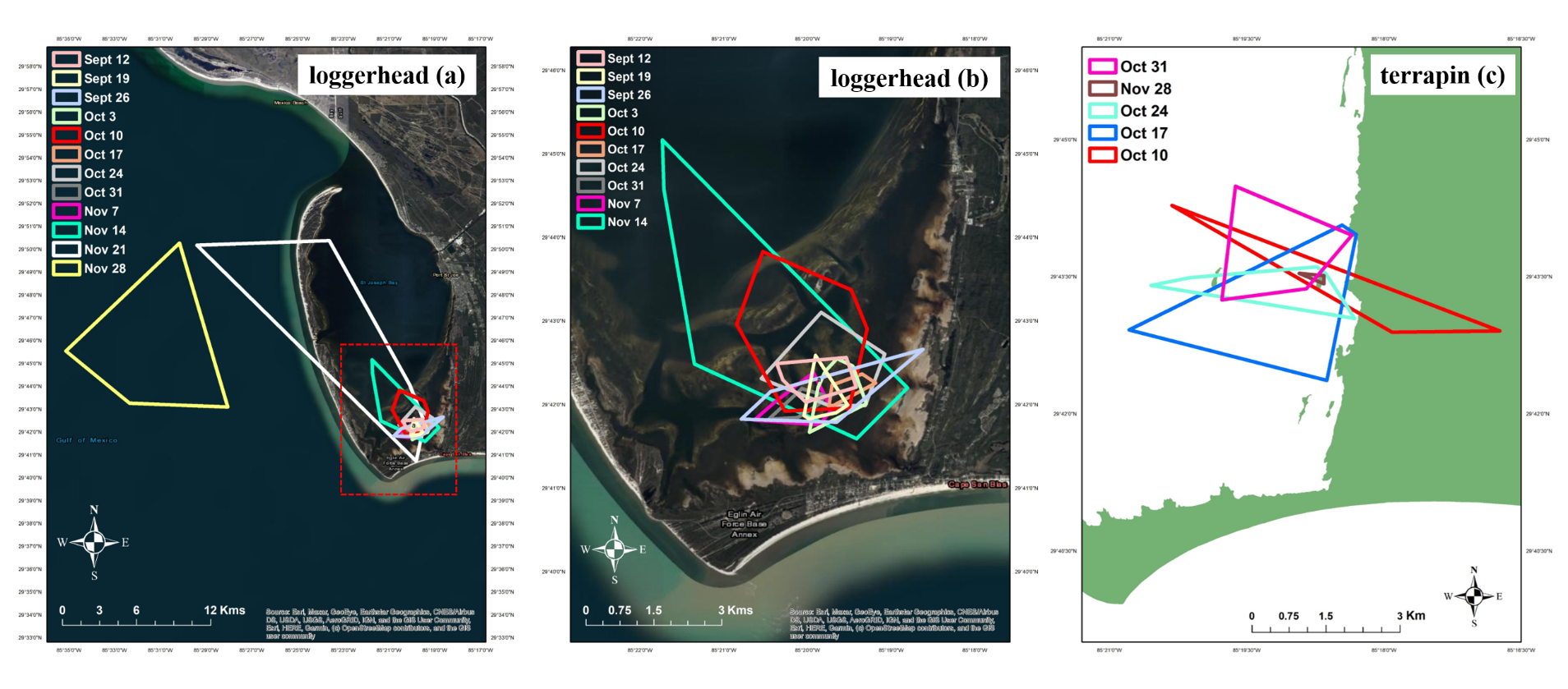


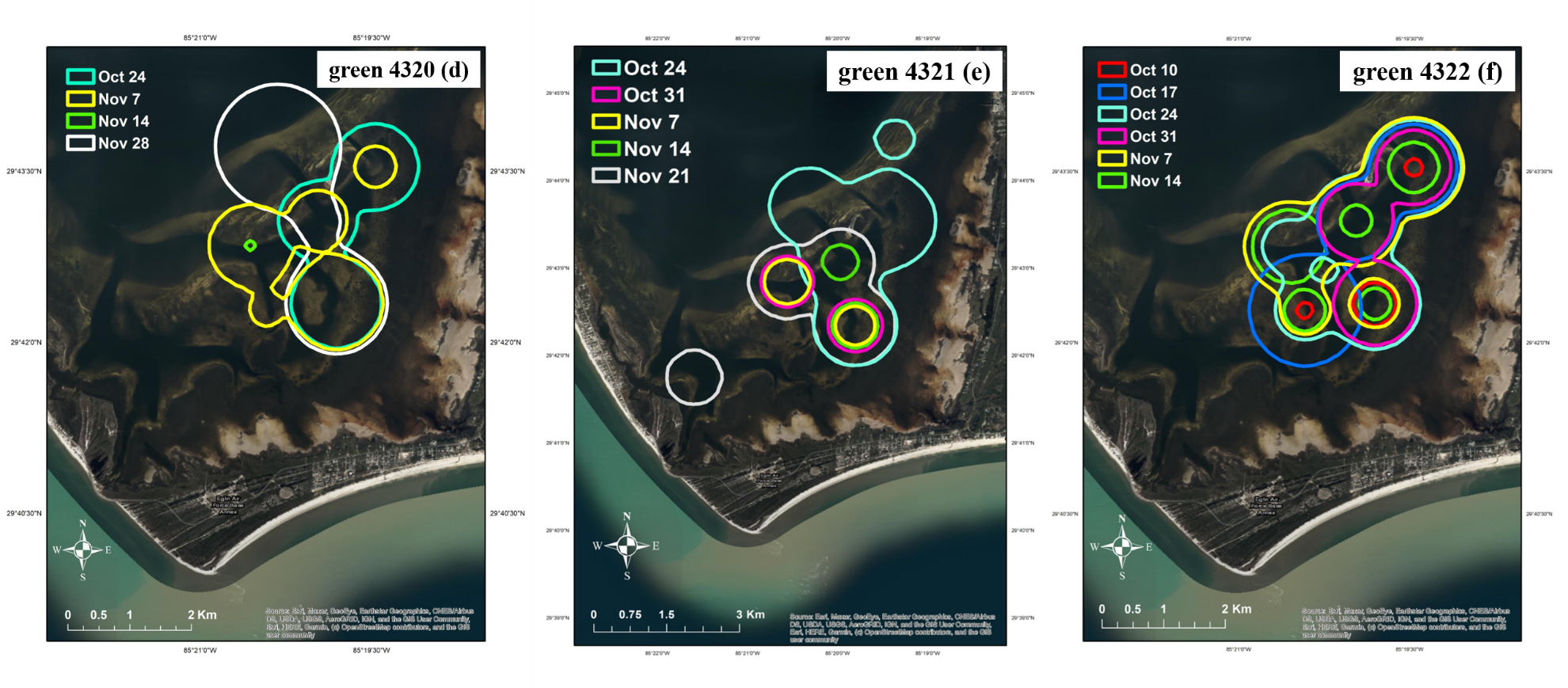


Supplementary Figure S1.
